# The Literature of the Pulp

**Published:** 1901-09

**Authors:** Vida A. Latham

**Affiliations:** Chicago, Ill.


### THE LITERATURE OF THE PULP.[1]

BY VIDA A. LATHAM, M.D., D.D.S., F.R.M.S., CHICAGO, ILL.

IN reviewing the work done on the dental pulp, it appeals very strongly to the operative side. In other words, the majority of the papers concern themselves with the *pathologic* conditions, which include special and general pathologic states from inflammatory to degenerative and bacteriologic gangrene to formation of pulp-stones and the treatment of the same. One noteworthy point is the constant effort directed to the "killing of the pulp" by every known means and the very little encouragement to try to save the "formative organ." In these days of advancement of the histo-physiologic research, and of the introduction of new methods and chemicals, it seems strange that the dental pulp appears to have been considered as either completely worked out or not so important an organ as enamel or dentine, or even the peridental membrane. Yet its development, structure, functions, and variations are hardly understood,—and we expect to cure its ailments! It is true a great deal has been investigated and many essays and papers presented from all parts of the world, but very little truly scientific work has been completed.

In this paper, so far as possible, references to the operative treatment of the pulp have been excluded, and in considering the anatomy, histology, and especially the development of the pulp many authors have described the neighboring structures of odontoblasts, dentine, and enamel, it being impossible to separate them wholly from the pulp. Any one wishing to study the latter must be willing to examine also the former.

Dentists are now waking to the fact that we must have more preliminary training in the elementary branches, and schools of dentistry are constantly improving. It would be a worthy object to found grants, scholarships, and opportunities for original research work to post-graduates or advanced workers, instead of such memorials as oil-paintings, busts, etc. The former would carry far more weight, would perpetuate memory in a nobler way, and reach to almost every clime and class by constant quotation,

---

[1] Abstract of a paper read before the American Medical Association, Section on Stomatology, at St. Paul, Minn., June, 1901.

thus enriching science and benefiting thousands of suffering humanity. How many libraries contain works on dentistry accessible to the student that are of recent date or valuable in looking up the subject? It has been said that publishers object to giving the large medical and scientific libraries a copy of a book for fear of lessening its sale. Personally, I think this objection should not be sustained, as many people who note a new book in a library or store, finding it suited to their needs, will buy a copy to have at hand. Owing to the scarcity of accessible works, even in the majority of our large central cities, the task of "looking up literature" is by no means easy or worthily done, oftentimes many essays and papers scattered through the numerous journals being out of reach. Better libraries are needed.

Perhaps the earliest mention of the dental pulp is by Galen,[1] who states: "He felt the pulsation in a tooth that was aching in the same way as any other inflamed part. I know that the seat of one pain was in the tooth and the other in the gum." To Galen we may also ascribe the discovery of soft nerves supplied to the teeth.

In 1771 John Hunter noted the building up and closing in of the pulp-cavity when attrition has worn the teeth, as did George Prochaska (1780) in his work published in 1800. Besides these, we may note Thomas Bell, Bertin Rousseau, Lent, Raschkow, R. Owen, Alexander Nasmyth, Kölliker, R. T. Hulme, E. Albrecht, R. Hohe, J. Bruck June, F. Ulrich, and many others whose papers chiefly relate to calcifications in the pulp and pathology.

About the earliest histologic point I have found so far seems to be by Paul B. Goddard,[2] who described the pulp as composed of granular matter invested by a delicate membrane or epithelium.

Professor L. S. Beale in 1865 states the pulp-tissue is not converted into dentine, neither does the dentine nor the tissue from "which it is formed exhibit any characters justifying in stating it to be connective in origin. Dentine is only produced by the agency of the so-called cells." The mass of the pulp is composed of a single form of connective tissue with numerous oval and triangular corpuscles (germinal matter), not unlike the mucous tissue of the umbilical cord. (This will seem to be foreign to the title of this paper, but I find in almost all articles the close relation of dentine and its histology and origin the chief topic.)

Kölliker[3] summarized the dental tissues in one of the first text-books which held its place for many years. The odontoblasts were formally termed "dentinal cells" (Elfenbeinzellen). Kölliker termed this entire layer of cells *Membrana eboris,* because after the pulp has been withdrawn it usually cleaves to the inner surface of the tooth in the form of a continuous membrane-like layer. Kölliker, Klein, and others state that odontoblasts excrete dentine, differing from Waldeyer, who has a most complete and satisfactory theory as regards the development of the teeth. He also makes mention of Sharpey's fibres being formed in the cementum, and agrees with Boll that the odontoblasts (a term originating with Waldeyer) send fibres and connect with each other by lateral processes. He also edits the article on "Formation of Dentine,"[4] which formed the basis of a controversy with Professor H. Hertz in Virchow's Archives, and was used by Franz Boll in order to explain the contradictions of the two investigators and so form an independent opinion.

Franz Boll is an investigator to whom we owe much. His work[5] is standard to-day, even in the light of so much new research by better methods and apparatus. Professor Waldeyer, of the University of Breslau, eminent as an histologist and microscopist, says in his famous essay on "The Mouth and Development of the Teeth," "We owe to Mr. Boll (a medical student at Bonn, where his essay was written) the first definite knowledge of the condition of the nerve-fibres in the teeth." Boll says we *never* secure the whole pulp or *all* the soft part of it, as the odontoblastic processes project into the tubes of the dentine. Let one be ever so careful in extracting the pulp from freshly cracked teeth, a trace of this peculiar layer will rarely be found on the surface of the pulp. But if cracked and then hardened in chromic acid solution for an hour, using considerable care in removing broken dentine and a sharp knife, one may sometimes obtain the superficial layer of the pulp *in continuo.* The peripheral processes which are enclosed in the dentinal tubes generally break close to the body of the cell, but by a skilful movement of the knife they may sometimes be successfully extracted of considerable length from the tubules.

J. Tomes described nerve-fibrils in the dentine continuous with the pulp net-work, and considered these fibrils to be the cause of sensitive dentine. Boll in his demonstrations used the long incisors of rodents, as guinea-pigs, rabbits, etc., which may make some difference as regards the attachment of the pulp to the dentine.

Goodsir's work [6] was received without question by most anatomists, if not by all, inasmuch as it gave a very definite intelligible description of observations in place of vague general notions, especially as regards the development of enamel, which was, in 1863, clearly demonstrated by Professor Kölliker.

Legros and Magitot, in their work,[7] give a very excellent *résumé* of much value. This work, published in 1873, was translated in 1880 by M. S. Dean, of Chicago.

J. Raschkow [8] mentioned that the teeth developed under the mucous membrane and enamel-organ. He described the blood-supply of the pulp by two or three arteries, and asserted that the pulp has one large and a few smaller nerves, the latter breaking up near the surface into an exceedingly fine plexus called the Plexus of Raschkow. Boll and others suggest that fine filaments are given off from this plexus which inosculate with the branches of the odontoblast cells, but as yet this connection has not been demonstrated and accepted as a fact.

Neumann,[9] in 1863, demonstrated the dentinal sheaths, Boll following out the dental nerves in their further course more recently.

Tomes [10] has most carefully and successfully continued the study of the finer points of dental structure, and, by first demonstrating the dentinal fibres, opened the way to a correct interpretation of the nature of dentine.

Lent [11] demonstrated the dentinal processes of odontoblasts.

Klein [12] says the cells beneath the odontoblasts are spherical and nucleated, and according to his view the dentinal process is derived from these cells, not from odontoblasts.

Retzius [13] observes, by the use of Golgi's stain, that the nerve-fibrils of the pulp appear to penetrate between the odontoblasts and terminate between them and the dentine.

Röse [14] says with increasing age the odontoblasts appear to suffer atrophic changes.

Erwin Höhl [15] states that three kinds of cells occur in the pulp at different life periods:

(1) The outermost peripheral layer of branched cells.

(2) Centralward the conjugation cell layer, containing the elementary or primary odontoblasts, the largest process of the cell becoming the future dentinal fibril.

(3) Secondary odontoblasts (dentinoblasts) by conjunction of the first and second layers.

R. R. Andrews [16] states that odontoblasts in forming the matrix of dentine give out minute globular bodies which form a layer of calcoglobulin, not being themselves any part of the matrix. The fibres within the tubes are formed from a separate cell, deeper in the pulp-tissue, whose processes pass in through the protoplasmic mass of the odontoblasts to reach the dentinal tubes of the formed dentine.

BLOOD-SUPPLY OF THE PULP.

Bödecker,[17] in discussing Mr. Witzel's work, says, "I have not been able to see an artery in a pulp as illustrated, though I will not say there may not be one." So far he has only met with capillaries. Arteries go into the pulp and divide at once upon entering it. In fœtal pulps a number of arteries are seen extending to the base of the tooth, but on entering are only seen as capillaries.

Dr. W. H. Atkinson [18] says no embryologist will state that there are three coats on the vessels in the pulp; the large vessels are sinuses.

Dr. A. O. Hunt [19] notes Dr. Patrick's demonstration of the methods of blood-supply to the follicle of the developing tooth. Specimens were prepared as follows: Injections of gelatin carmine were made through the carotid artery; sections cut from the jaw were put into absolute alcohol and ground down, no water being used, only alcohol to keep them wet. Examination of these specimens showed that the blood-supply of the tooth did not pass directly from the artery through the foramen, but was distributed through the cancellous bone by a number of small branches and all along the peridental membrane by a system of capillaries. He believed the nerves did not pass directly through the foramen, but by a circuitous route accompanying the blood-current. All the blood-vessels stopped on the outside of the membrane, and the supply was there taken up and distributed by a system of capillaries. A fœtal (eight months) tooth showed this clearly, and he thought it true of the developed specimen. He doubted the old theory that the artery and nerve-fibre entered the foramen directly, and he also thought the office of the peridental membrane was simply to sustain what it had built up. In other words, the artery breaks into capillaries, passes into the cancellous bone about the teeth, is distributed to the peridental membrane, and from it to the pulp through the apical foramen or a number of foramina in the root.

Black,[20] in discussing the above paper, agreed with Dr. Hunt in the statement that no direct branch from the dentinal artery enters the apical foramen and supplies the pulp, as depicted in so many text-books, but that many arteries, branches from the main dentinal arteries, are distributed to the bone of the alveolus and the peridental membrane, forming a net-work which gives off branches that enter the dental foramen and supply the pulp. "In children you will find a half-dozen, and before the root is fully formed you will find hundreds of them entering the pulp from all directions, but as the foramen decreases in size they are shut off until there is but one."

J. Leon Williams[21] describes the blood-supply of the pulp as coming from two sources,—viz., the pericementum and the medullary canal,—and has observed in recently developed teeth small blood-vessels passing from the pericementum through the side of the root into the pulp; also, in the odontoblast layer of cells, he notes an "intricate plexus of very fine blood-vessels in the odontoblastic layer of cells."

H. H. Burchard[22] believes (though he has not demonstrated) that the pulp has an extensive collateral circulation, the branches of the inferior dental arteries anastomosing freely with the branches of the external maxillary, facial, coronary, mental, submental, sub-lingual, gingival, internal maxillary and its branches, other than the inferior dental.

Cryer's dissections showed that in the lower jaw the inferior dental artery is the main supply.

In the embryo, as early as three months, a blood-supply in the dentinal papilla is evident, vessels showing in the dentinal papilla (the future pulp) before the follicular walls (in part the future pericementum) is outlined. "When the dental tissues proper begin to form, the follicles lie in a gutter of bone, their bases (the future necks of the teeth) separated from the bone by a comparatively thin layer of fibro-vascular tissue in which lie the inferior dental vessels. At this stage numerous arterial trunks are seen passing into the immature pulp." At seven months the lateral blood-supply (from the periosteum) is more marked than that from the base of the dentinal papilla and quite distinct from the vascular supply to the pulp. Bone forms around all the arterial trunks, enclosing them, the vessels being at first enclosed by indifferent tissue, this becoming fibrous and, finally, ossification taking place.

Hertwig's "Embryology" describes the visceral arches at an early stage in the development of the ovum, and states that differentiation of the tissues of the visceral arches into bone, muscle, fascia, and glands changes the anatomical relations of the arteries originally supplying the arches.

Burchard's conclusion is that the pericementum is supplied from arteries which enter the apical space in several branches from the dental arterial trunks and that there is free anastomosis with the vessels of the alveolar walls. The pulp is supplied by several trunks from the dental arteries. In case of obliteration of these trunks collateral circulation is established through the vessels of the pericementum and alveolar walls.

Retzius investigated the *endings of the pulp-nerves in fishes, reptiles, amphibians, and mammals.* He used the Golgi method and traced the nerve-fibres to their terminal fibrillæ, which appeared under the dentine or on the free surface of the pulp, occasionally, in the teeth of young mice, running between the odontoblasts and the dentine, but never in the dentinal tubules.

Huber [23] studied the *nerve-supply of the tooth-pulp in dogs, cats, and rabbits,* employing the *intra-vitam* methylene-blue method of staining. He demonstrated that the medullated nerve-fibres going to the pulp in large bundles break up into small bundles which anastomose and form a plexus in the fibrous tissue membrane covering the under surface of the pulp. From this plexus smaller bundles of medullated nerves pass perpendicularly into the pulp, and can be traced "to all levels of the pulp, some of them to the very tip." Besides these, many medullated fibres leave the bundles and approach the surface of the pulp near the lower surface of the odontoblastic layer, losing their sheaths or breaking up into branches which are non-medulated with varicosities and nuclei, and which branch and interlace with each other, forming a network under the odontoblasts. Terminal fibres given off from this net-work pass up between the odontoblasts and terminate in fine granules near the free ends of the odontoblasts. Somewhat rarely the fibres may be found crossing over the odontoblasts and ending between the odontoblasts and the dentine. In these findings Huber coincides with Retzius, and he states positively that he found no nerve-fibre in the dentine and no fibre connected directly with the odontoblasts or with any other cell-elements of the pulp. "The terminal fibrillæ or fine branches given off from them terminate in free endings between the odontoblasts or between these cells and the dentine, and are not in conection with the dentinal fibrils directly or through the odontoblasts."

Morgenstern [24] endeavors to establish principally certain special points:

(1) Innervation of the blood-vessels.

(2) Relation of the axis cylinder to various cellular components.

(3) The nerve-endings.

(4) Structure of the nerve plexus beneath and within the odontoblastic zone.

(1) The innervation is enormous. All the arteries, even the finest arterioles, exhibit an extremely fine and elaborate plexus. In the larger blood-vessels it shows a very regular arrangement of the fibres, one set running parallel with the blood-vessels and another at right angles to them. In this way each nucleus is made to rest, as it were, in a net-work of nerves. One or more axis cylinders are frequently found with the finest capillaries, not uncommonly embracing these in spirals. This was especially easy to prove in the crown pulp of a calf. Morgenstern found similar formations in dentine that had been treated by the corrosive sublimate method (Golgi), and had afterwards been colored with hæmatoxylin. The larger nerve-fibres have Ranvier cells at fairly regular intervals; the finer ones have few cells or none at all, although they have many knobby enlargements, which appear in the finest fibres only as points.

(2) Nerve-endings in the pulp are difficult to determine, as one can never assert with absolute assurance that where a nerve-fibre appears to terminate, it does not really continue in some other direction out of your field of observation. I believe they terminate as free fibres, especially when they divide like a brush into a number of very fine threads. Many axis cylinders end in small knobby enlargements, beyond which for the most part a very fine short thread extends.

(3) Between and below the odontoblasts the nerves frequently terminate in rounded cellular bodies which could be better shown by another method, by means of which they are brought into sharper contrast with their surroundings. These bodies are identical with the terminal disks described above, we assume. Whether the nerves extend throughout the entire odontoblastic zone—a most important question—could be answered positively by Bethe's modification of the methylene-blue process. In the so-called horns of the pulp, more correctly the crest, the nerve-fibrils are universally present between the elementary cells, which are but imperfectly united to form odontoblasts. These nerve-fibrils lie in close parallel lines and send out a large number of lateral branches between the elementary cells, which are side by side in rows. The fibres show by their marked blue tint, and by the fact that they spring from the primary nerve-fibres, that they are integral constituents of the nervous system which extends throughout the pulp. Dentine germs from human and animal fœtal jaws treated by the aniline-blue methods of Ciaglinsky and others (Stroebe) showed the fibrils of Weil's zone to be colored blue, like axis cylinders, but that the dentinogenous substance was likewise colored blue. Where odontoblasts had been formed there were often seen narrow transverse strips of blue tint,* by which the odontoblasts seemed to be divided into segments.

Erwin Höhl also saw this appearance, but correctly explained them as fibrils of the intercellular net-work of nervous elements, and this can be proved by the methylene-blue method and Nikiforoff. Morgenstern's formic acid was peculiarly efficacious. That cells which belong to the nervous system do occur between the odontoblasts is proved by preparations made with methods 2 and 6. The peculiar chemic and optic properties of the odontoblastic zone serve to explain why the morphologic constituents are so difficult to recognize both there and in the membrane præformativa. The nearer the odontoblastic zone approaches the dental process the more thoroughly does it seem to be filled with a hyaline substance which opposes an almost insurmountable obstacle to all investigations.

Whether ganglion cells occur in the pulp is perhaps answered by the methylene-blue test to the extent that remarkably large

---

* Entwicklungsgeschichte der Zähne in Scheff's Handbuch der Zahnheilkunde, Bd. i. S. 280, 281.

cells connected with at least two nerve-fibres occur, especially near the larger blood-vessels,—*i.e.*, in the axial portion of the pulp.

SUMMARY.

(1) The nerves of the pulp are divided into central and parietal nerves according to their location. The nerve-branches of the parietal system are more slender, but much more numerous than those of the central. They are spread in two directions, axial and radial, the former following the long axis of the tooth, the latter that of the dentine canals.

(2) Although belonging to the parietal system, its outermost stratum forms a system in itself.

(3) More strongly developed nerve-fibres of the central system form no proper plexus in the pulp; those of the parietal system form temporarily in the young pulps a plexus.

(4) Medullary sheaths of the pulp are secondary formations only determined positively in teeth that have been cut.

(5) Odontoblastic zone is traversed in the most varied directions by nerve-fibres.

(6) Nerve-fibres terminate in several ways: (*a*) Free in the pulp; (*b*) knobby enlargements with projecting supplementary fibre; (*c*) disk bodies frequently seen in the odontoblastic zone.

(7) In the central part of the pulp are some very large cells which may be considered as ganglion cells.

(8) Different varieties of endings occur even in the enamel. These remarkable results of Morgenstern's have been questioned by Röse, who thinks it a precipitate in the dentinal tubules and on the processes of the odontoblasts.

Dr. W. G. Atkinson Robertson[25] says the odontoblasts in the teeth of oxen are bipolar, one process being a dentinal fibre and the other continuous with the nerves of the pulp. He thus regards the odontoblasts and dentinal fibres as becoming in course of development the terminal organs of the nerves of the tooth. These facts and the development of the dentine were made on the teeth of rabbits and kittens, in the former taking advantage of the aid given by feeding the animal for a time on food containing madder, which stains the dentine matrix produced while the madder is being taken, just as it does the matrix of bone. The elongation of the dentine results from proliferation of connective-tissue cells in a formative ring at the base of the tooth-follicle. The thickening of the dentine results from new layers of matrix added from within under the influence of odontoblasts, not from interstitial growth, which produces nothing more than a slight increase in matrix between the dentinal tubules in the crown of the tooth. To the question whether the nerve passes as a main fibre from the trunk nerve direct through the foramen, or are the nerves in the pulp continuous with the nerves in the pericementum, statements have been made that if the nerves extended through the foramina the broken nerve would be seen extending beyond the end of the root, which is never the case. If any one will take the pains to examine teeth as soon as extracted, examples can often be seen with a projecting pulp or nerve (?) varying from one-sixteenth to one-eighth of an inch in length, sometimes even more. They appear as whitish threads coming from the apex, especially in cuspids, incisors, and bicuspids, bleached on account of the sudden rupture and hemorrhage. Unless noted at the time, the shrinkage is so great it might not be perceived, though carefully fixed material will show the pulp in some cases, specimens of which I have now.

### NERVE-SUPPLY OF THE PULP.

J. L. Williams emphasizes the vasomotor nerves of the pulp and pericementum, showing branches (non-medullated) distributed to the blood-vessels. He suggests that stimulation or injury of these nerves may paralyze the vasoconstrictor fibrils a short time before paralysis of the vasodilator fibrils occurs, thus explaining the engorgement of the vessels which strangulates the pulp in arsenical destruction of the latter. The origin of these vasomotor nerves, he states, is "in the upper jaw from the anterior palatine nerve, which, arising from the spheno-palatine ganglion, is connected with the sympathetic system through that branch of the Vidian nerve which arises from the carotid plexus; and in the inferior maxilla their connection with the sympathetic system is through a nerve branch from the facial artery which enters the submaxillæ ganglion, through the chorda tympani, and also the otic ganglion, from a branch arising from the plexus of the middle meningeal artery."

Burchard believes that the nerve-supply of the pulp is from the branches of the dental trunks proper, though admitting that nerves pass over the alveolar rim into the pericementum. In mature teeth he is not certain as to the point of entry. But in a figure showing a cuspid tooth at thirteen years the nerves one-third of the way up the canal are seen to consist of several bundles of medullated fibres which follow the course of the main blood-vessels, splitting into fibrillæ, "beneath the layer of odontoblasts, with which they have a doubtful relationship."

Black states that nerve-bundles distributed to the apical pericementum as well as to the pulp have a common origin,—namely, the inferior dental nerve in the lower jaw,—and that in the upper jaw blood-vessels and nerves together penetrate the antral wall "into channels of the basal portion of the alveolar bone."

In conclusion, let us note the great diversity of opinions prevailing among histologists. Are not these questions of vital importance before we can in any way reliably attempt or even venture to cure the pathologic conditions brought to our notice? How much more important is the histology, embryology, bacteriology, and pathology of the dental tissues compared to the subject of physics by lectures and laboratory, as is now being put in the fourth year of some of our dental colleges when it really belongs to the elementary chemic course. Let us encourage workers to give some time optional or obligatory during the senior or the post-graduate year, and give them better facilities by *money grants through the Association,* whose committee on special research could determine whether the candidate was worthy of support or not, and let the papers be published in the *Journal* if worthy of recognition.

BIBLIOGRAPHY.

[1] Galen, quoted by Von Georg von Carabelli, 1831-44; Systematisches Handbuch der Zahnheilkunde, 2d, vol. in Atlas in 4°. 8°. Wein.

[2] Goddard, Anatomy, Physiology, and Pathology of Human Teeth, Philadelphia, 1844.

[3] Kölliker, Gewebelehre, 5th Aufl., Mikros. Anat., 1854. Manual of Histology, 1853, annotated by Bush and Huxley.

[4] Waldeyer, Unteras über die Entwicklung der Zähne Abth., Königsberger Med. Jahrbücher, Bd. v., 1864; Anth. Zeitschr. Med. R. Jahrbücher, Bd. xxiv., 1865; Article on Structure and Development of the Teeth, Stricker's Histology, vol. i., New Sydenham Society, Pub.

[5] Boll, Archiv. für Mikros. Anatomie, vol. iv., Bonn, 1868; Remarks upon the Tooth-Pulp, Quarterly Jour. Micro. Sci.

[6] Goodsir, Origin and Development of the Pulp and Sacs of the Human Teeth, Edinburgh Med. and Surg. Jour., 1838.

[7] Legros and Magitot, Sur l'Origine et Formation du Follicle Dentaire, Jour. Anat. de la Phys., 1873, translated by M. S. Dean, 1880, Chicago.

[8] Raschkow, J., Die Gesundheitslehre des Mundes, 1835, Ludwigsburg.

[9] Neumann, Beiträge zur Kenntniss des Normalen Zahn und Knochengewebes, Leipzig, 1863.

[10] Tomes, Dental Anatomy and Physiology, 1848; System of Dentistry, Trans. by Zur Nedden, 1862; Rodents, London, Philadelphia, Trans. 1850; Marsupials, London, Philadelphia, Trans. 1849; On the Presence of Soft Tissue in the Dentinal Tubes, London, Philadelphia, Trans. 1846. P. ii.

[11] Lent, Beiträge zur Entwicklung des Schmelzes und Zahnbein; Zeitschr. für Mik. Zool., 1854, vi.

[12] Klein, Atlas of Histology and Elements of Histology.

[13] Retzius, Gustav, Zur Kenntniss der Nervenendigungen in den Zähnen. Biologische Untersuchungen Neue Folge, iv. p. 65, 1892; Ueber die Nervenendigungen in den Zähnen bei Amphibien. Biologische Untersuchungen Neue Folge, v. p. 40, 1893; Zur Kenntniss der Endigungsweise der Nerven in den Zähnen der Säugethiere. Biologische Untersuchungen Neue Folge, vi. p. 40, 1894.

[14] Röse, Dental Cosmos, vol. xxxv.

[15] Höhl, Erwin, Archiv. für Anat. in Entwicklung, Leipzig, 1896, pp. 31-54; Beiträge zur Hist. der Pulpa und das Dentin.

[16] Andrews, R. R., Dental Cosmos, 1891, p. 144; Text-Book of Operative Dentistry.

[17] Bödecker, Dental Cosmos, vol. xxi., p. 613; Catching's Compendium, 1896.

[18] Atkinson, W. H., Dental Cosmos, 1891, p. 144.

[19] Hunt, A. O., Dental Cosmos, 1890, pp. 32, 90, 905; also Illinois State Dental Society Transactions, 1891, pp. 73-76.

[20] Black, G. V., Trans. Illinois State Dental Society, 1891; Periosteum and Peridental Membrane, 1887.

[21] Williams, J. Leon, Dental Cosmos; Items of Interest, New York, 1898.

[22] Burchard, H. H., Text-Book Dental Pathology; Therapeutics; Items of Interest, New York, 1898.

[23] Huber, G. C., The Innervation of the Tooth Pulp, Dental Cosmos, 1898, p. 797.

[24] Morgenstern, Michael, Investigations of the Nerves of the Pulp by the Methylene-Blue Method, Abstract in INTERNATIONAL DENTAL JOURNAL, April, 1897; original article in Deutsche Monatschrift für Zahnheilkunde, September, 1896.

[25] Robertson, Dr. W. G. Atkinson, Meeting of the Scottish Microscopic Society, Edinburgh, December 19, 1890, Lancet.

